# The Role of Vasospasm and Microcirculatory Dysfunction in Fluoropyrimidine-Induced Ischemic Heart Disease

**DOI:** 10.3390/jcm11051244

**Published:** 2022-02-25

**Authors:** Natalia Fabin, Maria Bergami, Edina Cenko, Raffaele Bugiardini, Olivia Manfrini

**Affiliations:** Laboratory of Epidemiological and Clinical Cardiology, Department of Experimental, Diagnostic and Specialty Medicine, University of Bologna, 40138 Bologna, Italy; natalia.fabin@studio.unibo.it (N.F.); maria.bergami@unibo.it (M.B.); edina.cenko2@unibo.it (E.C.); olivia.manfrini@unibo.it (O.M.)

**Keywords:** cardio-oncology, cardiotoxicity, cancer, ischemic heart disease, fluoropyrimidines

## Abstract

Cardiovascular diseases and cancer are the leading cause of morbidity and mortality globally. Cardiotoxicity from chemotherapeutic agents results in substantial morbidity and mortality in cancer survivors and patients with active cancer. Cardiotoxicity induced by 5-fluorouracil (5-FU) has been well established, yet its incidence, mechanisms, and manifestation remain poorly defined. Ischemia secondary to coronary artery vasospasm is thought to be the most frequent cardiotoxic effect of 5-FU. The available evidence of 5-FU-induced epicardial coronary artery spasm and coronary microvascular dysfunction suggests that endothelial dysfunction or primary vascular smooth muscle dysfunction (an endothelial-independent mechanism) are the possible contributing factors to this form of cardiotoxicity. In patients with 5-FU-related coronary artery vasospasm, termination of chemotherapy and administration of nitrates or calcium channel blockers may improve ischemic symptoms. However, there are variable results after administration of nitrates or calcium channel blockers in patients treated with 5-FU presumed to have myocardial ischemia, suggesting mechanisms other than impaired vasodilatory response. Clinicians should investigate whether chest pain and ECG changes can reasonably be attributed to 5-FU-induced cardiotoxicity. More prospective data and clinical randomized trials are required to understand and mitigate potentially adverse outcomes from 5-FU-induced cardiotoxicity.

## 1. Introduction

In recent years, important progress in the oncological field has led to an increase in cancer survival rates in high-income countries [1]. Cardiovascular toxicity is not uncommon, with more and more oncological patients having to co-exist with cardiovascular comorbidities. This has led to the concept of “cardio-oncology” [2,3].

Fluoropyrimidines, such as 5-fluorouracil (5-FU) and its oral prodrug capecitabine, are the third most common drug used for the treatment of solid malignancies such as breast, colorectal, head, neck, gastrointestinal, and bladder cancer [4,5,6]. These antimetabolite drugs have a structure similar to that of the substrates and enzymes necessary for DNA replication; specifically, 5-FU is an analogue of uracil with a substitution by fluorine of a hydrogen at position 5′ of the pyrimidine ring [7,8]. Cytotoxicity results from 5-FU inhibiting the activity of thymidylate synthase (TS) and from misincorporating its metabolites into DNA and RNA [9,10]. Capecitabine is a prodrug converted to 5-FU through thymidine phosphorylase, an enzyme highly present inside tumor cell. This results in higher intratumor concentrations of 5-FU compared with normal tissue [11].

Regrettably, fluoropyrimidines are strongly associated with cardiac adverse events and are the second most common drug associated with cardiotoxicity after anthracyclines [6]. The clinical manifestations of fluoropyrimidines-induced cardiotoxicity are various, ranging from chest pain, hypotension, or dyspnea to myocardial infarction, life-threatening arrhythmias, and death [6,12].

Although the pathophysiological mechanisms behind fluoropyrimidines-induced cardiotoxicity are yet to be fully understood, coronary vasospasm and microcirculatory dysfunction most likely play a role in the development of myocardial ischemia and chest pain, which is the predominant manifestation of 5-FU induced cardiotoxicity.

This review aims to examine the characteristics and the role of vasospasm and the potential role of microcirculatory dysfunction in patients with fluoropyrimidines-induced ischemic heart disease. The included studies consisted of both prospective and retrospective studies (Table 1).

## 2. The Quality of Chest-Pain Symptoms

The most common clinical manifestation of 5-FU cardiotoxicity is chest pain, which can be either nonspecific or anginal and is often, but not always, associated with electrocardiographic (ECG) changes [26] (Table 1). Chest pain may occur at rest or be effort related. Serum biomarkers of cardiac injury are rarely elevated, as documented by some observations. ECG evidence of ischemic ST-T changes can be recorded in 69% of patients, but abnormal cardiac enzymes can be found in only 12% [51]. A recent article has differentiated between a more typical and acute chest pain and a more atypical and persistent chest pain, both due to fluoropyrimidines [52]. A 42-year-old woman developed a crushing chest pain across her precordium during the first bolus of 5-FU. The ECG showed diffuse ST-segment elevation, but coronary angiography showed no epicardial coronary artery disease (CAD). In contrast, a 54-year-old man experienced a typical retrosternal chest discomfort at rest. ECG changes were undetectable. Coronary computed tomography angiography was performed with no evidence of epicardial CAD. Both patients were diagnosed as having coronary vasospasm. These case reports well illustrate the variety of chest pain symptoms at presentation that may accompany 5-FU cardiotoxicity, which may constitute a great source of consternation to practicing physicians. What, therefore, are the primary implications of this finding? First, although many studies have well documented that patients are more likely to experience subsequent coronary events if they present with typical symptoms [53], it is recommended that clinicians do not exclude the occurrence of coronary vasospasm based only on the quality of the symptoms. Second, these data do not suggest that all patients presenting with non-specific chest pain require specific treatment for coronary vasospasm even if they have recurrence of the symptoms. Conversely, these data reinforce the idea that diagnosis of coronary vasospasm would be missed in most patients if clinicians rely on the credence that symptoms should fit with the description of classic angina pectoris. Further investigations, including 24 h ECG monitoring, angiography, or computed tomography angiography, are required.

## 3. Risk Factors for Chest Pain Due to Fluoropyrimidines

Several potential risk factors for 5-FU chest pain have been suggested, including underlying CAD, older age, and concomitant use of other treatments with cardiac side effects. Patients with pre-existing cardiac disease were at elevated risk of cardiotoxicity (risk ratio = 6.83, *p*-value = 0.0023) in a small cohort in which cardiotoxicity occurred in 7 of the 209 patients receiving their first course of 5-FU [27]. Nevertheless, there have been some inconsistencies with such hypotheses, as documented by further studies including patients who underwent coronary angiography for persisting angina. Coronary angiography did not show obstructive CAD in any of these patients [28]. Older age was supposed to be a risk factor for 5-FU chest pain. However, data did not support this belief [29,51]. Concomitant administration of other chemotherapeutic agents with cardiac side effects has been suggested as a reason for an increased risk of 5-FU cardiotoxicity. Still, this is a further assumption as there was only some evidence for increased cardiotoxicity with concomitant cisplatin treatment. The effects of previous or current chest-radiotherapy were also ambiguous [30].

In summary, to date there are insufficient data to ascertain risk well enough to justify withholding therapy in patients undergoing 5-FU treatment.

## 4. The Long-Standing History of Fluoropyrimidines-Induced Vasospasm

Cases of angina or myocardial ischemia following administration of 5-FU or capecitabine have been reported since the early 1970s [54]. Several case reports and case series suggested vasospasm as one of the possible mechanisms leading to ischemia of the myocardium (Table 1). This hypothesis is mainly based on the improvement of symptoms after administration of nitrates or calcium channel blockers (CCBs), as well as on the absence of angiographic evidence of obstructive CAD. Other indirect evidence of vasospasm comes from the clinical characteristics and ECG findings of patients administered with fluoropyrimidines. The observation of effort angina pectoris or angina at rest accompanied by transient ST-segment elevation, mainly recorded during the recovery phase of stress testing, are all features compatible with an acute, transient, complete obstruction of the coronary flow rather than with a chronic flow-limiting coronary stenosis [36,55]. In the early 1990s, Luwaert and colleagues [37] actually documented focal coronary vasospasm in a 70-year-old man after two boluses of 5-FU. Over the following years, studies conducted on animal models reinforced the role of vasospasm as a plausible pathophysiologic mechanism leading to myocardial ischemia: Mosseri and colleagues observed increasing rates of endothelium-independent vasoconstriction in rabbit aorta rings following administration of progressively higher dosages of 5-FU [56]. Such observations were confirmed in human models, where 5-FU exerted a higher vasoconstricting effect on brachial arteries of treatment arm compared with control [57]. Südhoff and colleagues attributed a leading role in the pathophysiology of fluoropyrimidine-induced cardiotoxicity to vasoconstriction. This hypothesis was supported by the observation that coronary vasoconstriction occurred during or immediately after the infusion of 5-FU, disappeared after drug discontinuation, and reoccurred when further cycles of 5-FU administration were given. Some observations of vasospasm and vasospasm-induced myocardial ischemia were also made after administration of capecitabine [38].

## 5. Uncertainties in Vasospasm Characterization and Its Impact on Management

Despite the clear clinical and pathophysiologic importance of vasospasm as a putative mechanism of fluoropyrimidine-related cardiotoxicity, this side effect is yet to be fully characterized (Table 1). Indeed, most studies investigating this issue included small, heterogenous samples that did not undergo routine coronary angiography during the onset of symptoms [39,40]. Chest pain and ECG findings cannot establish a firm diagnosis of coronary vasospasm. In cancer patients, the perception of chest pain is altered [58], either by a direct effect exerted by the tumour itself or by the administration of analgesics and narcotics prescribed to treat cancer pain, such as opioids in combination with non-steroidal anti-inflammatory drugs [59] or acetaminophen [60]. The lack of a clear symptomatology suggestive of coronary vasospasm in oncological patients has been confirmed by Rezkall and colleagues [31]. These authors conducted a prospective study on 25 patients undergoing 5-FU infusion. Following continuous ECG monitoring, 17 patients had asymptomatic ECG changes during infusion. It follows that the real epidemiology of coronary vasospasm following fluoropyrimidines administration is complex and needs to be fully understood.

Incidence of coronary vasospasm in 2021, a large cohort study by Zafar and colleagues [32], aimed to better define the real incidence of fluoropyrimidine-induced vasospasm. The study comprised 4019 patients who received either bolus or infusion therapy with 5-FU. Of these patients 87 (2.6%) developed coronary vasospasm, mostly after the first cycle of therapy. These patients were younger (age 58 ± 13 years vs. 64 ± 13 years; *p* = 0.001) and had fewer cardiovascular risk factors (70.1% vs. 84.5%; *p* = 0.007) when compared to those not developing coronary vasospasm. No sex- or race-dependent differences were observed between the two groups. Although in the analysis by Zafar and colleagues, no differences in prognosis were observed between patients with and those without coronary vasospasm, other investigations have observed patients with of severe outcomes, ranging from development of myocardial infarction to sudden cardiac death [51]. 

The risk of coronary vasospasm depends on the cumulative dose [41], the schedule [33], and the route of administration [34] and it is increased in the presence of a concomitant administration of cisplatin-based chemotherapy which leads to hypomagnesemia [61]. Re-challenge is not advised since there is a higher risk of death, myocardial infarction, and cardiogenic shock [42]. If re-challenge is needed, the American College of Cardiology recommends switching to a bolus regimen rather than continuous infusion. Further, it recommends administering nifedipine and isosorbide mononitrate before treatment, short-acting diltiazem and sublingual nitroglycerin during the treatment, and the association of nifedipine with isosorbide 12 h after the treatment. Finally, it recommends administering only nifedipine 24 h after the treatment [62].

## 6. Focal or Diffused Coronary Spasm: An Important Distinction

Coronary vasospasm is not a homogeneous entity. The term “vasospastic angina” refers to a condition that may be caused by focal coronary artery spasm or by diffuse spasm of the entire coronary arterial tree, namely vasotonic angina. These two major categories are distinct entities and should be managed differently [63].

### 6.1. Focal Coronary Spasm

Focal epicardial coronary artery spasm may be identified in some patients with angiographically normal or near normal coronary arteries. Focal epicardial coronary artery spasm is often considered a synonymous of variant angina, also called Prinzmetal’s angina. However, it should be recognized that coronary artery spasm is not specific to Prinzmetal angina. On selective coronary angiography, 0.5–0.8% of patients could show evidence of coronary artery spasm stimulated either by the tip of the catheter or the contrast medium [64]. Focal epicardial coronary vasospasm can be effectively detected through intravenous administration of ergonovine or acetylcholine [65,66]. It is characterized by local segmental coronary hyperreactivity of the smooth muscle to a variety of stimuli that produce only mild constriction in non-spastic segments of the coronary arteries [67]. Focal coronary vasospasm is usually found in correspondence of mild atherosclerotic plaques. Atherosclerotic disease affecting large coronary arteries altered their vasomotor tone and reactivity. There is an intimate association of spasm with sites of organic stenosis. Atherosclerotic segments may be deficient in the production of prostacyclin, which has marked endothelial-dependent vasodilation and platelet aggregation inhibiting properties. This could result in unopposed effects of thromboxane A2 released by platelets and subsequent vasoconstriction and activation of platelet glycoprotein IIb/IIIa receptor [68].

### 6.2. Diffuse Coronary Spasm: Vasotonic Angina

Diffuse spasm is a manifestation of endothelial dysfunction that alters and enhances vessel reactivity to normal sympathetic stimulation, partly due to reduced shear-mediated nitric oxide (NO) release and excess of reactive oxygen species [69,70]. A heightened direct constrictor response of vascular smooth muscle can also be implied [71]. This disorder may affect the epicardial coronaries as well as the microcirculation. Coronary microcirculation includes different anatomically and functionally vascular compartments (<500 µm diameter) and has a critical role in the physiological regulation of myocardial perfusion [72]. Since it cannot be seen in invasive procedures such as angiography, its role is often just an assumption.

A study conducted by a research group of the University of Bologna is crucial to demonstrate that vasotonic angina actually is a disorder of the entire coronary arterial tree [66,73]. Patients with vasotonic angina showed epicardial vasoconstriction which was usually severe and confined to the distal segments of the coronary arteries. Despite administration of intracoronary nitroglycerin and the resolution of spasm in the epicardial arteries, the researchers found that the resistance to blood flow was persisting, which suggests that the microcirculation is still the major culprit. It has been estimated that coronary microvascular spasm accounts for approximately 27% of patients with myocardial infarction with non-obstructive coronary artery disease (MINOCA) [72,74].

### 6.3. Type of Coronary Spasm Associated with Fluoropyrimidine Administration

In the case of fluoropyrimidine-induced cardiotoxicity, several case reports and case series reported clinical pictures compatible with variant angina in patients who had a history of angiographically documented coronary artery lesions [43]. However, invasive assessment of the coronary tree was often not repeated after fluoropyrimidine administration, so a definite causal association between coronary atherosclerotic plaques and coronary vasospasm could not be ascertained. In 1989, Kleinman and colleagues [39] described the case of a 63-year old man treated with 5-FU who presented with recurrent episodes of angina followed by transient ST-segment elevation that was promptly relieved by nitrates administration, and effectively controlled by the use of CCBs. These elements induced the authors to hypothesize that the patient may have developed Prinzmetal’s angina. Still, no angiographic testing was conducted to document the occurrence of coronary spasm. In a study by Luwaert and colleagues [37], the type of coronary spasm detected at angiography appeared to be focal, with a 70% narrowing of the left circumflex artery, a degree of vasoconstriction that may induce chest pain even in the absence of significant ST elevation. Other investigators, however, were unable to uncover focal spasm during ergonovine testing [44]. Moreover, recent reports described cases of capecitabine-induced diffuse spasm accompanied by clinical and echocardiographic signs of Takotsubo Syndrome [45].

The evidence of fluoropyrimidine-induced diffuse spasm of the coronary arterial tree is compliant with the hypothesis that endothelial dysfunction or primary vascular smooth muscle dysfunction (an endothelial-independent mechanism) is a possible contributing factor to this form of cardiotoxicity. Fluoropyrimidines exert their toxic effect on the endothelium through a plethora of different mechanisms. Cwikiel and colleagues [75] observed that these antitumor agents caused direct cytotoxic effect on endothelial cells, as demonstrated by the high level of vessel wall and endothelial cell contraction, cell edema, cytolysis, occurrence of denuded areas, platelet adhesion/aggregation and fibrin formation. These factors certainly act through endothelial nitric oxide synthase (eNOS) [76], as NO produced by eNOS and its interaction with serine/threonine protein kinase Akt/PKB [77], is a key determinant of cardiovascular tone [78,79,80]. These factors have also been demonstrated to increase the levels of free radicals and endothelin-1, which has a potent vasoconstricting effect [81].

### 6.4. The Role of Microcirculation

Microvascular dysfunction appears to be involved in fluoropyrimidine-induced coronary vasospasm. This hypothesis is supported by the evidence of global akinesia or dyskinesia at echocardiography in myocardial regions not supplied by stenotic coronary arteries [82]. However, this finding must be interpreted cautiously in light of a recent report [83]. The combination of endothelial dysfunction (defined as a decrease in luminal diameter of >20% after intracoronary acetylcholine) and microvascular dysfunction (defined as an index of microcirculatory resistance of ≥25) is present in only 10% of the overall population of patients with normal or near-normal coronary arteries. Indeed, the majority of these patients were found to have myocardial bridging (55%), and a substantial number of patients had some evidence of atherosclerosis based on intravascular ultrasound examination. It follows that microvascular dysfunction and atherosclerosis, although causally related in many patients, are distinct problems and may exist separately. Thus, many patients may show myocardial ischemia subsequent to hidden atherosclerosis, normal coronary arteries at angiography, and normal microvascular function. Coronary thromboemboli, without causing significant obstruction, could induce coronary artery spasm and cause acute myocardial infarction [84]. Such thromboemboli could occur mainly in patients with prosthetic valves or cancer [85,86].

## 7. Type of Spasm and Impact on Therapy

Focal coronary artery spasm can be effectively treated by CCBs and nitrates [43,46,47,48,86]. The renin–angiotensin system inhibitors may prove to be useful in long-term management as well. Treatment of diffuse coronary spasm is less defined, with a possible role for angiotensin-converting enzyme inhibitor therapy and supplementation with folic acid, which may alleviate the impaired endothelium-dependent arterial vasodilation. Of note, some studies were at variance and showed that in a few patients with variant angina the administration of L-type CCBs (nifedipine, verapamil, and diltiazem) or nitrates were not successful in relieving symptoms [35,49,50,56]. This finding may support the possible role of microcirculation at least in some patients, as the microvessels have a greater prevalence of T-type calcium channels, and L/T-type CCBs (e.g., mibefradil and efonidipine) have a higher efficacy compared with L-type CCBs [87]. The positive role of L/T-type CCBs on the microcirculation has been shown by studies that described clinical and angiographic improvements in the coronary slow flow phenomenon following the administration of mibefradil [88] or nicardipine [89]. 

A further caveat should be noted. Shimokawa et al. [90] demonstrated that as the vessel size of the coronary microcirculation decreases, there is a progressive major role of endothelium-derived hyperpolarizing factor (EDHF) rather than nitric oxide, the latter being more important in the vasodilatation of epicardial large vessels.

In summary, the heterogeneity in the response to CCBs and to nitrates could explain why there are variable results after administration of CCBs or nitrates in some patients with 5-FU presumed to have myocardial ischemia. Induced chest pain persisted after the administration of some types of CCBs or nitrates. One point is critical. Testing that separates those patients whose symptoms are due to myocardial ischemia from those whose pain is non-ischemic is strictly necessary. Even minimal atherosclerotic disease on angiography (or intravascular ultrasound imaging) warrants risk-factor modification and prevention therapies.

## 8. Management of Cardiotoxicity

Cardiotoxicity related to 5-FU administration is a poorly understood but relatively common clinical entity that deserves special consideration given the frequent use of this agent and the cardiac complications associated with its use. The mechanism of 5-FU-related cardiotoxicity may occur from a combination of ischemia related to epicardial coronary vasospasm and microvascular dysfunction. Further clinical studies in humans are required to clarify these mechanisms. Patients with 5-FU based chemotherapy who develop vasospasm are difficult to manage. Patients who present with chest-pain symptoms should be routinely referred to cardio-oncology for optimization of their medications. Patients should receive a baseline ECG. Patients should also be instructed to stop therapy and to call the emergency department if experiencing any chest discomfort at home. Clinicians should determine if further 5-FU is required or whether acceptable alternative treatments can be safely used. When further doses of 5-FU are necessary, clinicians should proceed cautiously. Management of the affected patients should focus on separating patients whose symptoms are due to myocardial ischemia from those whose pain is non-ischemic. Coronary angiography or computed tomography angiography is required as it would seem prudent to perform a sensitive screen for significant fixed coronary disease prior to performing any provocative testing for a diagnosis of coronary spasm. Patients with normal or near-normal coronary arteries may undergo ergonovine or acetylcholine tests, which would result in a better characterization of the vasospastic disease [91]. The possible coexistence of coronary spasm in patients with severe coronary disease is clinically irrelevant. Patients that have severe “fixed” coronary disease at angiography need revascularization. Old, but still current guidelines support the usefulness of provocative testing for coronary spasm in patients with “recurrent episodes of apparently ischemic cardiac pain at rest” and “normal or mildly abnormal coronary angiogram” but no clinical observations, such as ST segment shifts during rest ECG or 24 h ECG monitoring to substantiate the diagnosis of variant angina [92]. It should not go unnoticed, however, that physicians have abandoned the use of intracoronary provocative testing in the last two decades. Some cardiologists believe that ex juvantibus criteria such as the use of CCBs, possibly combined with nitrates, may be helpful as provocative testing in the diagnostic evaluation of spasm. We may argue that chest pain with or without ECG shifts is not synonymous of variant angina. Coronary spasm in variant angina is a distinct entity and should be managed with CCBs and nitrates. Symptom-relieving drugs, such as beta blockers, have been found to be effective in patients with vasotonic angina and/or microvascular dysfunction [93]. Even minimal atherosclerotic disease warrants risk-factor modification and prevention therapies. Avoiding unnecessary medicines and optimizing therapy when linked to the correct diagnosis will benefit patients, health care providers, and the health care system.

## 9. Conclusions

In summary, the evidence of coronary vasospasm and microcirculatory dysfunction in 5-FU-induced ischemic heart disease is still lacking. Physicians would consider urgent coronarography for patients presenting with suspected acute coronary syndrome and computed tomography angiography for low-risk patients with persistent minor or mild symptoms. Additional provocative tests should be performed in patients with unobstructed coronary arteries. Such an approach permits discrimination of variant angina and vasotonic angina along with coronary microvascular disorders versus non-cardiac chest pain, which permits distinct treatments outlined in consensus practice guidelines. When further doses of 5-FU are required, clinicians should proceed cautiously and wait for a definitive diagnosis of coronary artery disease.

## Figures and Tables

**Table 1:** Summary of studies evaluating the incidence of coronary vasospasm in patients treated with 5-FU.

Author [Reference]	Sample Size, *n*	Study Design	Fluoropyrimidine Type	Overall Incidence of 5-FU Induced Cardiotoxicity, %	Presentation of Cardiotoxicity	Coronary Angiography/Ventriculography
Polk et al. [13]	2236	Retrospective	5-FU or capecitabine	4.6%	Chest pain Myocardial infarction Cardiac arrest Sudden death Heart failure	Angiography performed in 24 patients: 13% had obstructive CAD Coronary spasm was not documented at coronary angiography
Polk et al. [14]	452	Retrospective	5-FU	4.9%	Chest pain Myocardial infarction Ischemic ECG changes (ST-deviation followed by T waves abnormalities) ECG changes or arrhythmias (atrial fibrillation, QTc prolongation) Dyspnoea Cardiac arrest	N/A
Abdel-Rahman et al. [15]	3223	Pooled analysis of five RCT	5-FU	7.9%	Chest pain Myocardial infarction ECG changes or arrhythmias (Atrial flutter, Atrial fibrillation, AV-block, BBB, Ventricular arrhythmias)	N/A
Peng et al. [16]	527	Retrospective	5-FU or capecitabine	30.6%	Chest pain Myocardial infarction Ischemic ECG changes (ST-deviations/ T wave abnormalities) ECG changes or arrhythmias (atrial fibrillation, conduction blocks) Heart failure	N/A
Kwakman et al. [17]	1973	Pooled analysis analysis of three RCTs	Capecitabine	5.9%	Chest pain Myocardial injury/infarction Myocardial infarction ECG changes or arrhythmias (Atrial fibrillation, AV block, Ventricular fibrillation) Heart failure	N/A
Tsibiribi et al. [18]	1350	Prospective	5-FU	1.2%	Chest pain Myocardial infarction	N/A
Akhtar et al. [19]	100	Prospective	5-FU (Continuous infusion)	8%	Chest pain ECG changes Cardiogenic shock	N/A
Keefe et al. [20]	910	Prospective	5-FU	0.55%	Chest pain Myocardial infarction ST-elevation Ventricular arrhythmias Cardiac arrest	N/A
de Forni et al. [21]	367	Prospective	5-FU (Continuous infusion)	7.6%	Chest pain Unstable angina Ischemic ECG changes (ST-deviation; T-wave inversion) Sudden death Arrhythmias Dyspnea	N/A
Jeremic et al. [22]	80	Prospective	5-FU and cisplatin	15%	Chest pain Ischemic ECG changes (ST-T wave abnormalities) Arrhythmias	N/A
Eskilsson et al. [23]	76	Prospective	5-FU (Continuous infusion) and cisplatin	18%	Chest pain ECG changes or arrhythmias (Atrial fibrillation, ventricular fibrillation) Sudden death	N/A
Labianca et al. [24]	1083	Retrospective	5-FU	1.6%	Chest pain Myocardial infarction	N/A
Pottage et al. [25]	140	Prospective	5-FU	2.9%	Chest pain ST segment deviation; T-wave inversion Myocardial Infarction	N/A
Ng et al. [26]	153	Pooled analysis of two prospective trials	Capecitabine and oxaliplatin	6.5% (Chest pain 4.6%)	Chest pain at rest Chest pain during exertion Elevated troponins Ischemic ECG changes (ST-depression, Q waves, T waves abnormalities) ECG changes or arrhythmias (Ventricular tachycardia/fibrillation) Sudden cardiac death Heart failure	One patient
Meyer et al. [27]	483	Prospective	5-FU (Continuous infusion)	1.9%	Chest pain ECG changes or arrhythmias (bradycardia, tachycardia, RBBB, PVCs) Hypotension Hypertension Dyspnea	N/A
Wacker et al. [28]	102	Prospective	5-FU (80% continuous infusion; 20% bolus)	19%	Chest pain with ECG changes Ischemic ECG changes (ST-deviation) ECG changes or arrhythmias (bradycardia, PVCs, Prolonged QTc)	Six patients: All non-obstructive CAD Coronary spasm was not documented at coronary angiography
Jensen et al. [29]	668	Retrospective	5-FU or capecitabine	4.3%	Chest pain	N/A
Khan et al. [30]	301	Retrospective	5-FU	19.9%	Chest pain Elevated biomarkers of myocardial necrosis Ischemic ECG changes (ST-deviation, T wave changes) ECG changes or arrhythmias (Bradycardia, AV block, Ventricular tachycardia) Cardiac arrest Hypotension Hypertension Heart failure	N/A
Rezkalla et al. [31]	25	Prospective	5-FU (Continuous infusion)	24%	Chest pain at rest during infusion Ischemic ECG changes (ST-deviation during infusion) Sudden death	N/A
Zafar et al. [32]	4019	Retrospective	5-FU	2.2%	Chest pain Elevated troponins (conventional or high sensitivity) Ischemic ECG changes (ST-deviation, T wave changes) Dyspnea Syncope	N/A
Kosmas et al. [33]	644	Prospective	5-FU and oral capecitabine	4.03%	Chest pain/discomfort Ischemic ECG changes (ST-deviation, T wave changes) with or without raised biomarkers of myocardial necrosis ECG changes or arrhythmias (PVCs, AV blocks) Malaise, diaphoresis Syncope	N/A
Meydan et al. [34]	231	Prospective	5-FU (2 days infusional regimen)	3.9%	Unstable angina Myocardial infarction Pericarditis Congestive heart failure Atrial fibrillation	N/A
Eskilsson et al. [35]	58	Prospective	5-FU	14%	Chest pain Ischemic ECG changes (ST-segment elevation followed by T-wave inversion) ECG changes or arrhythmias (Ectopic atrial rhythm, Prolonged PR interval)	N/A
**Case reports and case series**						
Lestuzzi et al. [36]	3	Case report	5-FU (Continuous infusion)	100%	Chest pain during effort with ECG changes (ST-elevation/-depression; negative T waves)	N/A
Luwaert et al. [37]	1	Case report	5-FU (Continuous infusion)	100%	Chest pain at rest, with ST-segment elevation in leads I.aVL, V4-6, and II,III,aVF	Non-obstructive CAD Coronary spasm was not documented at coronary angiography
Henry et al. [38]	1	Case report	Capecitabine	100%	Chest pain during effort	N/A
Kleiman et al. [39]	1	Case report	5-FU (Continuous infusion)	100%	Chest pain with ECG changes (ST segment elevation) and PVCs	N/A
Suresh et al. [40]	1	Case report	5-FU (Continuous infusion)	100%	Chest pain at rest during infusion	Non-obstructive CAD Coronary spasm was not documented at coronary angiography
Frickhofen et al. [41]	1	Case report	5-FU (Continuous infusion)	100%	Chest pain with ECG changes (negative T-waves in leads AVL, I and V4–V6), unresponsive to treatment Recurring chest pain with ECG changes (ST- elevations in leads I, II, AVL, AVF and V3–V6), unresponsive to treatment	Non-obstructive CAD Coronary spasm was not documented at coronary angiography
Clasen et al. [42]	11	Case series	5-FU	100%	Persistent chest pain and ischemic ECG changes (ST- elevation) Intermittent and recurrent chest pain	4 patients: 3 patients had evidence of non-obstructive CAD; 1 patient had evidence of flow limiting stenosis on RCA requiring stent apposition 3 patients had coronary CT scans with no evidence of CAD or coronary calcification Coronary spasm was not documented at coronary angiography
Alter et al. [43]	1	Case report	5-FU and cisplatin	100%	Chest pain during infusion of 5-FU relieved by treatment discontinuation Evidence of ischemia in septal, infero-septal and in the inferior wall on SPECT	Non-obstructive CAD Diffuse spams of the circumflex artery on cold pressor test during angiography reversed by coronary vasodilators
Arbea et al. [44]	1	Case report	Oxaliplatin and oral capecitabine	100%	Chest pain at rest Chest pain with ECG and stress echocardiography abnormalities (ST-elevation in precordial and inferior leads and akinesia and severe hypokinesia in the territory of the RCA and LAD)	Non-obstructive CAD No evidence of inducible epicardial vasospasm during ergonovine testing
Klag et al. [45]	1	Case report	Capecitabine	100%	Acute chest pain Elevated troponins ST-elevation Dyspnea	Non-obstructive CAD Apical dyskinesia with typical apical ballooning and systolic dysfunction Acetylcholine-induced diffuse vasospasm of the LAD, reversed by coronary vasodilators
Kim et al. [46]	1	Case report	5-FU (Continuous infusion)	100%	Acute chest pain ST-elevation in lateral leads	Significant atherosclerosis in the proximal left circumflex artery requiring DES apposition Coronary spasm was not documented at coronary angiography
Yuan et al. [47]	2	Case series	5-FU	100%	Chest pain Raised biomarkers of myocardial necrosis Ischemic ECG changes (hyperacute T waves, new LBBB) Left ventricular EF≤25% with severe hypokinesia Dyspnea	Coronary CT revealed normal coronaries with no stenosis.
Yildirim et al. [48]	1	Case report	5-FU (Continuous infusion)	100%	Chest pain ST-depression	N/A
Patel et al. [49]	7	Case series	5-FU	100%	Chest pain Ischemic ECG changes (new Q waves, ST-elevation/depression) Ventricular tachycardia Cardiac arrest Hypotension Left ventricular dysfunction	N/A
Akpek et al. [50]	1	Case report	5-FU (Continuous infusion)	100%	Recurrent chest pain during infusions relieved by treatment discontinuation and vasodilators Ischemic ECG changes (ST-elevation followed by T-wave inversion)	Normal coronary arteries at coronary angiography Coronary spasm was not documented at coronary angiography

Abbreviations: 5-FU = 5- fluorouracil; AV = atrioventricular; BBB = Bundle branch block; CAD = coronary artery disease; CT = computed tomography; ECG = electrocardiography; EF = ejection fraction; LAD = left anterior descending artery; LBBB = left bundle branch block; PVCs = premature ventricular contractions; RBBB = right bundle branch block; RCA = right coronary artery; SPECT = single photon emission computed tomography.

## Data Availability

Not applicable.
